# Transcriptional regulation by nonclassical action of thyroid hormone

**DOI:** 10.1186/1756-6614-4-S1-S6

**Published:** 2011-08-03

**Authors:** Lars C Moeller, Martina Broecker-Preuss

**Affiliations:** 1 Department of Endocrinology and Division of Laboratory Research, University of Duisburg-Essen, Hufelandstr. 55, 45147 Essen, Germany

## Abstract

**Abstract:**

Thyroid hormone (TH) is essential for normal development, growth and metabolism. Its effects were thought to be principally mediated through triiodothyronine (T3), acting as a ligand for the nuclear TH receptors (TRs) α and β residing on thyroid hormone response elements (TREs) in the promoter of TH target genes. In this classical model of TH action, T3 binding to TRs leads to recruitment of basal transcription factors and increased transcription of TH responsive genes.

Recently, the concept of TH action on gene expression has become more diverse and now includes nonclassical actions of T3 and T4: T3 has been shown to activate PI3K via the TRs, which ultimately increases transcription of certain genes, e.g. HIF-1α. Additionally, both T3 and thyroxine (T4) can bind to a membrane integrin, αvβ3, which leads to activation of the PI3K and MAPK signal transduction pathways and finally also increases gene transcription, e.g. of the FGF2 gene. Therefore, these initially nongenomic, nonclassical actions seem to serve as additional interfaces for transcriptional regulation by TH. Aim of this perspective is to summarize the genes that are currently known to be induced by nonclassical TH action and the mechanisms involved.

## TH and gene induction by classical mechanisms

**Figure 1:**
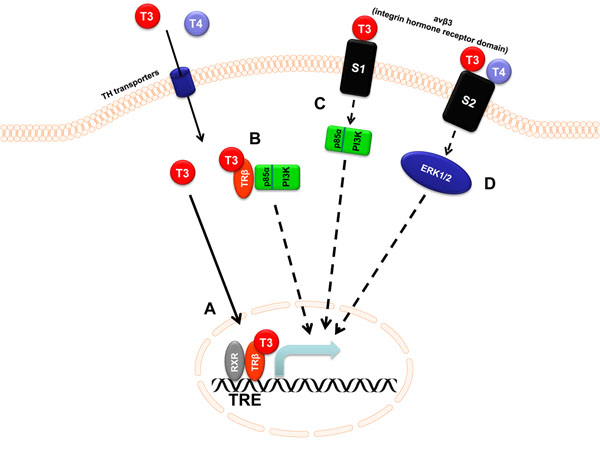
**Mechanisms of gene induction by nonclassical action of TH (modified from **[23,36]). A, classical TR/TRE mediated induction of gene expression; B, activation of PI3K by liganded TRβ; C, activation of PI3K via αvβ3/S1; D, activation of ERK1/2 via αvβ3/S2

Thyroid hormones (THs) are essential for normal development, growth, and metabolism, especially during fetal development and early childhood. In adults, the primary effects of THs are manifested by alterations in metabolism, including changes in oxygen consumption, protein, carbohydrate, lipid, and vitamin metabolism. The pleiotropic effects of THs on many different pathways and target organs become obvious by the clinical features of hypothyroidism and hyperthyroidism. TH action is mainly understood as modification of gene expression, mediated by the nuclear TH receptors (TRs) α and β as ligand dependent transcription factors. In the classical model of gene induction by TH, the TRs are located on thyroid hormone response elements (TREs) in the promoter of target genes, often as homodimers, but also as heterodimers with the retinoid-X receptor (RXR) [1,2]. In the unliganded state, corepressors are bound to the TR complex. Upon T3 binding, TR homodimers dissociate in favor of heterodimer formation. The corepressors are released and replaced by coactivators. This new complex ultimately engages the RNA polymerase II in transcription of the gene (Fig. 1 A).

This model also explains the dominant negative effect of a mutant TRβ on the wildtype TR in the syndrome of resistance to thyroid hormone (RTH). In RTH, patients have raised serum TH and raised or inappropriately normal TSH levels. Common clinical features of RTH include goiter, tachycardia, delayed bone growth with a variable phenotype [3]. In the majority of cases, RTH is caused by heterozygous TRβ gene mutations. One mutant TRβ as part of a homo- or heterodimer on a TRE could lead to reduced T3-binding, decreased corepressor release or decreased ability to bind coactivators. As a consequence, this TRE is blocked by a transcriptionally inactive TRβ complex and transcription of its gene is impaired despite the presence of normal TRβ encoded by the wildtype allele. The mutant TRβ thus confers a dominant negative effect on the wildtype receptor and consequently inhibits TH action [4]. An exception to this model is the index family described by Refetoff et al. with autosomal recessive inheritance [5] due to a homozygous deletion of the coding region of the TRβ gene [6]. Heterozygous members of this family were not affected clinically or biochemically, demonstrating that one wildtype copy of TRβ is sufficient for normal function. Until now, no mutations in the TRα gene have been described [2].

## Non-classical TH action

In the last ten years, large scale microarray studies have been performed to determine the effect of TH on gene expression in animal and human cells [7-12]. In recent years, it has become evident, that TH can act through various mechanisms: besides the classical TR/TRE-mediated mechanism (Fig. 1 A) these are the nonclassical mechanism of PI3K activation by either liganded TRβ (Fig. 1 B) or the integrin αvβ3 (Fig. 1 C) as well as activation of the MAPK cascade via αvβ3 (Fig. 1 D) (see below). All these mechanisms potentially influence gene expression. The initial step of pathway activation by TH is nongenomic, but the consequences include increased transcription of certain genes independent of TREs, which then are nonclassically induced TH target genes. While microarray studies helped defining gene expression changes in response to TH, their results represent a sum effect of both the classical TRE-dependent and the nonclassical pathway initiated mechanisms of TH action. It now seems necessary to attribute changes in gene expression to their underlying mechanisms. In this overview we aim to summarize which gene induction could be connected to nonclassical action of TH, especially in human cells (summarized in table 1).

**Table 1:** Gene induction attributed to nonclassical action of TH.

Gene	Proposed initial TH action	Method	Cell/tissue type	TH	TH concentration	time of TH tx	Ref.
ZAKI4α (RCAN2)	TRβ/PI3K	microarray/real time PCR/Western	primary human skin fibroblasts	T3	2 nM	24 h	[13,9]
HIF-1α a)	TRβ/PI3K	microarray/real time PCR/Western	primary human skin fibroblasts	T3	2 nM	24 h	[28]
HIF-1α b)	αvβ3(S1)/PI3K and αvβ3(S2)/ERK1/2	RT-PCR	U-87 MG	T3 / T4	1 nM / 100 nM	24 h	[23]
PFKP	TRβ/PI3K, target gene of HIF-1α	microarray/real time PCR/Western	primary human skin fibroblasts	T3	2 nM	24 h	[28]
GLUT1	TRβ/PI3K, target gene of HIF-1α	microarray/real time PCR	primary human skin fibroblasts	T3	2 nM	24 h	[28]
MCT4	TRβ/PI3K, target gene of HIF-1α	microarray/real time PCR	primary human skin fibroblasts	T3	2 nM	24 h	[28]
STC-1	TRβ/PI3K, target gene of HIF-1α	microarray/real time PCR	primary human skin fibroblasts	T3	2 nM	24 h	[31]
MCL1	TRβ/PI3K	RT-PCR	HK2, HEK293	T3	100 nM, free	6-24h	[32]
FGF2	MAPK	RT-PCR	ECV304	T4	100 nM	6-48 h	[33]

free = serumfree medium

### TRs and PI3K activation

One important nonclassical action of TH is the activation of PI3K via the liganded TR (Fig. 1 B). T3 had been shown to be able to activate the PI3K pathway with downstream phosphorylation and activation of PKB/Akt, mTOR and p70^S6K^[^13^]. This effect of T3 was initially observed as stimulation of Na,K-ATPase and KCNH2 activity that could be inhibited by the PI3K inhibitors LY294002 (LY) and wortmannin [14,15]. The crucial role of the TRβ for activation of PI3K became evident in experiments by Cao et al. in human skin fibroblasts [13]. Co-immunoprecipitation of both TRβ and the regulatory subunit of PI3K (p85α) demonstrated the association of TRβ and PI3K. Interestingly, the association of TRβ and PI3K is independent of T3, as could be shown in human skin fibroblasts transfected with wildtype TRβ or with the dominant negative mutant TRβ G345R that lacks T3-binding property [13] and with native TRβ in the rat pituitary cell line GH_4_C_1_[16]. But activation of the PI3K/mTOR pathway occurs only after T3 binding because in cells transfected with the TRβ G345R mutant this pathway was not activated by T3 [11]. Transfection of a dominant negative PI3K p85α regulatory subunit (Δp85α) expectedly also abrogated the T3 effect on the kinases downstream of PI3K, namely Akt, mTOR and p70^S6K^[13]. Further proof for PI3K activation by TRβ comes from a RTH mutant TRβPV, which constitutively activates PI3K [17].

Unliganded mutant TRβ is still able to bind to p85α without activating it. The markedly reduced effect of T3 on gene expression in the presence of a mutant TRβ in RTH is probably due to fact that an already reduced amount of liganded wildtype TRβ has to compete with unliganded mutant wildtype TRβ for p85α.

Similar results as for the TRβ were obtained for the TRα. Cao et al. could demonstrate PI3K activation by T3 mediated by TRα in TRα overexpressing neuronal cells [18] and Hiroi et al. found TRα1 association with the p85α subunit of PI3K followed by phosphorylation of Akt and activation of endothelial nitric oxide synthase [19].

### TH and the integrin αvβ3 receptor

In 2005, Davis and colleagues identified a structural plasma membrane protein, the integrin αvβ3, as a TH receptor that activated ERK1/2 [20]. Several lines of evidence supported this finding: radiolabeled T4 and T3 could bind to the purified αvβ3 protein, which was prevented by antibodies against αvβ3 as was ERK1/2 activation. SiRNA knockdown of either αv or β3 or both inhibited T4-induced MAPK activation. As several other integrins, αvβ3 contains a recognition site for the peptide sequence Arg-Gly-Asp (RGD). Pretreatment with an RGD peptide reduced ERK1/2 activation by T4 in the african green monkey fibroblast cell line CV-1. Tetraiodothyroacetic acid (tetrac), a deaminated T4 derivative, also blocked T4 action through the integrin [20]. Furthermore, effects of T4 were reproduced by T4-agarose, a modified thyroxine that does not cross the cell membrane [21]. Subsequent studies dissected sites and mechanisms of αvβ3 activation and led to the characterization of two different TH receptor sites, denoted S1 and S2, with different characteristics and intracellular consequences [22].

The S1 site of αvβ3 binds only T3 and is activated at physiological concentrations. This leads to phosphorylation and activation of Src and subsequently PI3K (Fig.1 C). T3 action on this site can be blocked by both tetrac and RGD, as demonstrated in human glioblastoma cells (U-87 MG): pretreatment with either RGD peptide or tetrac before addition of T3 abolished Src and PI3K phosphorylation [23]. Treatment with T3 leads to nuclear accumulation of TRα in a dose dependent manner. This was not observed after pretreatment with the PI3K inhibitor LY.

The other TH binding site on integrin αvβ3, S2, binds both T4 and, to a lesser extent, T3. TH binding to αvβ3/S2 then activates the ERK1/2 pathway (Fig. 1 D). Both T4 and T3 action can be inhibited by tetrac, but only T4 action by RGD peptide [23]. Results of TH binding to the S2 site of αvβ3 are increased angiogenesis [20], proliferation of glioma cells [24] papillary and follicular thyroid cancer cells [25] and TRβ shuttling from cytosol to the nucleus [23].

## Genes nonclassically induced by TH

### ZAKI4α

Activation of PI3K by TRβ/T3 and subsequent phosphorylation of downstream protein kinases such as Akt and p70^S6K^ leads to gene induction (Fig. 1 B) and transcriptional regulation by TH via this pathway could be shown for several genes. ZAKI-4α (also RCAN2 or DSCR1L1), a calcineurin inhibitor, for example, was already established as a thyroid hormone target gene in human primary fibroblasts but no canonical TRE was found in its promoter [26].

Induction of ZAKI-4α was attributed to the TRβ/PI3K-mechanism of TH action, because the T3 effect on ZAKI-4α was abrogated by pretreatment with PI3K-inhibitors wortmannin and LY294002 and transfection of Δp85α [13]. Additionally, an intact TRβ is required for ZAKI-4α induction. ZAKI-4α induction by T3 was abrogated after transfection of a dominant negative TRβ mutant (G345R) in human fibroblasts [13]. This was further supported by a blunted response to T3 in fibroblasts from patients with RTH either due to a heterozygous TRβ mutation or homozygous TRβ deletion compared to that in normal fibroblasts [9]. Pretreatment with cycloheximide (CHX) to prevent translation abrogated the T3 effect on ZAKI4α mRNA increase, demonstrating the requirement of prior *de novo* protein synthesis. In time course experiments, ZAKI4α mRNA accumulation in human primary fibroblasts was significantly increased only after 6 to 12 hours of T3 treatment [9,26]. ZAKI4α is therefore indirectly induced by TH and the mediating transcription factor, representing the immediate link to the PI3K pathway, has yet to be determined.

### HIF-1α

Among the genes found to be induced by T3 in a microarray study in human primary fibroblasts was Hypoxia inducible factor (HIF)-1α [9], one subunit of the basic helix-loop-helix transcription factor HIF-1. HIF-1 is a key mediator of angiogenesis and metabolic adaptation to hypoxia in tumors. It is responsible for elevated expression of glycolytic enzymes and glucose transporters. The HIF-1β subunit is constitutively expressed, while HIF-1α is tightly regulated [27]. HIF-1α synthesis is regulated by both PI3K and MAPK pathways, which themselves are activated by receptor tyrosine kinases or G-protein-coupled receptors. Ligands for these include growth factors and hormones such as growth factors and hormones EGF, IGF-1, insulin and androgens. T3 treatment in physiological concentrations was also found to increase HIF-1α mRNA and protein levels. T3 induced HIF-1α in normal fibroblasts, but not in fibroblasts from patients with RTH due to a TRβ mutation (A317T), indicating that the TRβ is required for this induction. Pretreatment with the PI3K inhibitor LY abrogated the T3 effect on both mRNA and protein, whereas inhibition of the MAPK pathway by PD98059 (PD) had no effect in primary human skin fibroblasts [28]. These results indicate that HIF-1α induction by T3 is mediated by TRβ/PI3K, similar to ZAKI4α. Other than for ZAKI4α, CHX pretreatment did not prevent HIF-1α mRNA increase. HIF-1α is therefore directly induced without the need for prior protein synthesis [28]. Whether this points to direct induction of HIF-1α mRNA synthesis or to activation of constitutively expressed transcription factors needs to be studied.

Lin et al. found HIF-1α to be induced in αvβ3-expressing human glioblastoma cells after treatment with T3 in physiological concentrations [23]. This effect was abrogated by pretreatment with LY. From these results the authors concluded, that T3-induced HIF-1α expression could be due to PI3K activation via the αvβ3/S1 site. Yet, specific data supporting that T3/PI3K-mediated HIF-1α induction is initiated at the integrin TH receptor, such as reduced T3 effect on HIF-1α expression after pretreatment with tetrac or RGD peptide, were not provided.

Interestingly, Davis and colleagues also observed a 1.5-fold HIF-1α mRNA increase after T4 stimulation in U-87 MG cells, an effect that seemed to be inhibited by PD pretreatment [23], which raises the possibility that HIF-1α induction could also be mediated by αvβ3/S2/ERK1/2.

### HIF-1 target genes

Besides the α-subunit of the transcription factor HIF-1, several of its known target genes, harboring hypoxia-response-elements in their promoter regions, were induced by TH in the same microarray study: glucose transporter 1 (GLUT1), phosphofructokinase (PFKP) and monocarboxylate transporter 4 (MCT4) [9]. Their products have important roles in cellular glucose metabolism: glucose uptake (GLUT1), glycolysis (PFKP) and lactate export (MCT4). The response of GLUT1, PFKP and MCT4 to TH with reproducible mRNA increase after physiological doses of T3, abrogation of this response by pretreatment with LY and lack of a significant response in cells with a mutant TRβ matches the pattern observed for HIF-1α and expected for HIF-1α target genes. In addition to the increase in mRNA, for PFKP an increase could be demonstrated for the protein by western blot. In contrast to HIF-1α, the induction of the PFKP, GLUT1 and MCT4 genes was inhibited by pretreatment with CHX, an inhibitor of protein synthesis. This requirement of prior protein synthesis is expected if their induction is mediated by HIF-1 [28].

Stanniocalcin (STC)-1 is a polypeptide hormone and was originally identified as a regulator of calcium/phosphate homeostasis in fish and mammals. In humans, STC1 has been implicated in angiogenesis, apoptosis and carcinogenesis and, recently, SUMOylation [29]. STC1 was recently characterized as a HIF-1 target gene as well [30]. STC-1 expression is inducible by T3, an effect dependent on TRβ and prior protein synthesis, and inhibited by LY and Δp85α. STC-1 therefore also belongs to this set of genes indirectly induced by TH via TRβ/PI3K and HIF-1 [31].

### MCL1

Myeloid cell leukemia (MCL)1 is an anti-apoptotic member of the B-cell lymphoma 2 (Bcl-2) family of apoptosis-regulating proteins. A twofold increase in MCL1 mRNA and protein was observed after treatment with 100 nM T3 in human kindey-2 (HK2) cells [32]. In subsequent promoter studies in HEK293 cells transfected with a luciferase reporter vector containing parts of the MCL1 promoter, treatment with 100 nM T3 in serumfree medium lead to a twofold increase in MCL1 promoter activity measured by a luciferase assay. In HEK293 cells, transcription from the MCL1 promoter also increases twofold in the presence of TRβ1, but not TRα1. Transcriptional activation seems not to be mediated by a potential TRE-DR4, because it also occurs in the presence of a disrupted TRE-DR4. This effect was abolished by inhibiting PI3K with wortmannin. These results indicate that MCL1 can be induced by T3 and that activation of PI3K and the TRβ1 are required, compatible with mechanism B (Fig. 1). From a dose response curve, T3 appears to have a significant effect on the MCL1 promoter only with doses of 100 nM and higher. These concentrations are about 100-fold physiological, and rather even higher, considering that serumfree medium instead of medium supplemented with T3-depleted serum was used. This and the fact that the results on the induction mechanism are from promoter studies limit conclusions towards the physiological relevance.

### FGF2

Finbroblast growth factor (FGF)2 is a member of the fibroblast growth factor (FGF) family and possesses angiogenic activities. In ECV304 cells, Davis et al. observed an increase in FGF2 RNA after stimulation with 100 nM T4 by RT-PCR [33]. FGF2 cDNA accumulation was significantly increased after 6 h and remained so for additional 42 hours. Treatment with T3 was not tested. In the same cells, T4 stimulated FGF2 secretion, measured by ELISA. This was inhibited by pretreatment with PD. Interestingly, treatment with 10 nM T3 also significantly increased FGF2 secretion and its effect was also inhibited by PD. These results can be interpreted as pointing to induction of FGF2 by T4 and T3 via the S2 site of the integrin TH receptor followed by MAPK activation (Fig. 2D), but involvement of both MAPK and αvβ3 in FGF2 gene expression still need to be determined specifically.

## Conclusion

TH can act through the TRs α and β on TREs, leading to gene induction (classical action), or act through TRs/PI3K in cytoplasm and nucleus or at the cell membrane on the integrin TH receptor αvβ3 (nonclassical action). These nonclassical actions of TH can lead to truly nongenomic effects, for example activation of the Na^+^/K^+^-ATPase. TH action via αvβ3 or TR/PI3K can also induce gene expression, e.g. for the genes describe above. These actions therefore constitute nonclassically initiated genomic actions of TH.

Results from most microarray studies so far delivered the sum of genes induced or repressed by TH without distinction of the mechanisms involved. For our own microarray study of T3 induced gene expression in human fibroblasts [9], we later found several of the most strongly induced genes after TH treatment to be downstream of TRβ/PI3K. Our interpretation was that in the PI3K signal transduction pathway the TH signal can be amplified from kinase to kinase (e.g. PI3K>AKT>mTOR) before reaching the genomic level. Amplification of TH action on a TRE seems limited in comparison.

While it is rather easy to measure TH induced gene expression, it seems much more complicated to pin down which of the possible mechanisms of TH action is responsible for the induction. Studies on nonclassical TH action often suffer from limitations and attribution to pathways and receptors is circumstantial. The connection between pathway and gene is not always complete, as for ZAKI the putative transcription factor linking PI3K and ZAKI4 expression is unknown. Furthermore, results from cells treated with excessive T3 concentrations need to be validated with more physiologic concentrations. The requirement of the TH receptor (TR or αvβ3) is not always convincingly shown. Nonclassically induced gene expression by TH would ideally be demonstrated by direct induction of a gene with use of physiologic TH concentrations, which is abrogated by both inhibiting the pathway and the initial TH receptor suspected to be involved. Ultimately, the connection of mechanisms of TH actions, sets of genes and (patho-) physiology is probably much more intricate than the rather simplistic descriptions here suggest. This is demonstrated by HIF-1α induction, for which three nonclassical mechanisms of TH action are possibly responsible (TRβ/PI3K, αvβ3/S1/PI3K and αvβ3/S2/ERK1/2).

Further studies of these newly described ways through which TH can modify gene expression may offer new therapeutic possibilities. The groups of Davis, Mousa and Hercbergs provided first examples and demonstrated that tetrac, which inhibits TH action at αvβ3, reduces growth of medullary thyroid cancer cells and renal cancer cells in mouse xenograft models [34,35].

## List of abbreviations

BTEB1: basic transcription element binding protein 1; CHX: cycloheximide; DSCR1L1: down syndrome critical region gene 1-like 1; ERK: extracellular signal-regulated protein kinase; FGF2: fibroblast growth factor 2; GLUT1: glucose transporter 1; HIF-1: hypoxia inducible factor 1; KCNH2: potassium voltage-gated channel: subfamily H (eag-related), member 2; LY: LY249002; MAPK: Mitogen-activated protein kinase; MCL1: myeloid cell leukemia 1; mTOR: mammalian target of rapamycin; PD: PD98059; PFKP: phosphofructokinase, platelet; PI3K: phosphatidylinositol 3-kinase; RCAN2: regulator of calcineurin 2; RTH: resistance to thyroid hormone; RXR: retinoid X receptor; STC1: stanniocalcin 1; T3: triiodothyronine; T4: thyroxine; tetrac, tetraiodothyroacetic acid; TH: thyroid hormone; TR: thyroid hormone receptor; TRE: thyroid hormone response element.

## Competing interests

The authors declare that no competing interests exist.
